# Inflammation of the choroid plexus in progressive multiple sclerosis: accumulation of granulocytes and T cells

**DOI:** 10.1186/s40478-020-0885-1

**Published:** 2020-02-03

**Authors:** Sabela Rodríguez-Lorenzo, Julia Konings, Susanne van der Pol, Alwin Kamermans, Sandra Amor, Jack van Horssen, Maarten E. Witte, Gijs Kooij, Helga E. de Vries

**Affiliations:** 1 Department of Molecular Cell Biology and Immunology, MS Center Amsterdam, Amsterdam Neuroscience, AmsterdamUMC, Vrije Universiteit Amsterdam, De Boelelaan, 1117 Amsterdam, The Netherlands; 2 Department of Pathology, MS center Amsterdam, Amsterdam UMC, Vrije Universiteit Amsterdam, Amsterdam, The Netherlands; 3 Centre for Neuroscience and Trauma, Blizard Institute, Barts and the London School of Medicine and Dentistry, Queen Mary University of London, London, UK; 4 Medical Biochemistry, Amsterdam Cardiovascular Sciences, Amsterdam UMC, University of Amsterdam, Meibergdreef 9, Amsterdam, 1105 AZ The Netherlands

**Keywords:** Choroid plexus, Progressive MS, Immune cells, T cells, Granulocytes, Blood-CSF barrier

## Abstract

The choroid plexus (CP) is strategically located between the peripheral blood and the cerebrospinal fluid, and is involved in the regulation of central nervous system (CNS) homeostasis. In multiple sclerosis (MS), demyelination and inflammation occur in the CNS. While experimental animal models of MS pointed to the CP as a key route for immune cell invasion of the CNS, little is known about the distribution of immune cells in the human CP during progressive phases of MS. Here, we use immunohistochemistry and confocal microscopy to explore the main immune cell populations in the CP of progressive MS patients and non-neuroinflammatory controls, in terms of abundance and location within the distinct CP compartments. We show for the first time that the CP stromal density of granulocytes and CD8+ T cells is higher in progressive MS patients compared to controls. In line with previous studies, the CP of both controls and progressive MS patients contains relatively high numbers of macrophages and dendritic cells. Moreover, we found virtually no B cells or plasma cells in the CP. MHCII+ antigen-presenting cells were often found in close proximity to T cells, suggesting constitutive CNS immune monitoring functions of the CP. Together, our data highlights the role of the CP in immune homeostasis and indicates the occurrence of mild inflammatory processes in the CP of progressive MS patients. However, our findings suggest that the CP is only marginally involved in immune cell migration into the CNS in chronic MS.

## Introduction

Multiple sclerosis (MS) is a heterogeneous disease of the central nervous system (CNS) characterized by immune cell infiltration, demyelination and neurodegeneration [24]. The most common clinical form of MS is relapsing-remitting MS (RRMS), in which disease exacerbations are followed by periods of relative inactivity and recovery. The majority of RRMS patients eventually evolve into a progressive phase called secondary progressive MS (SPMS). In some patients, however, MS is progressive from the onset, referred to as primary progressive MS (PPMS). In both forms of progressive MS, clinical symptoms mostly reflect the underlying neurodegeneration. The pathological processes involved in the different phases of MS are relatively well defined. In RRMS, there is abundant immune cell invasion into the CNS through a dysfunctional and inflamed blood-brain barrier (BBB), leading to inflammatory white matter lesions. In progressive MS, neurodegeneration becomes more prominent and inflammation subsides, although lesion activity is still present [21]. While immune cell infiltration through the BBB is reduced in progressive MS [9, 20], inflammatory processes at the other CNS barriers, such as those at the choroid plexus (CP) and meninges, may still contribute to the influx of peripheral immune cells. Indeed, it is known that chronic inflammation occurs in the meninges during progressive MS [6, 22, 26], but less is known about the choroid plexus (CP) immune populations in progressive MS patients.

The CPs are secretory tissues strategically located within the CNS. They are the main producers of cerebrospinal fluid (CSF) and therefore essential for regulation of CNS homeostasis. The CPs are located in each of the brain ventricles and consist of highly vascularized stroma surrounded by a tight continuous layer of epithelial cells. The vasculature of the CPs is characteristically fenestrated, resulting in a leaky interphase between the blood and the CP stroma. The tight junctions that connect the epithelial cells restrict the entry of molecules and cells into the CSF. As such, the epithelial cell layer in the CP is a pivotal component of the blood-CSF barrier (BCSFB). The BCSFB allows for a tightly regulated bidirectional immunosurveillance system in which immune cells can traffic through the CP into the CNS, but also vice versa [28]. Thus, the CPs, together with the BBB and the meninges, act as regulatory barriers for immune cells between the periphery and the CNS [4, 28].

Immune cells populate the CP under normal conditions, but a detailed overview of immune cell subsets that reside in the CP is currently lacking. The abundance of MHCII+ cells in the CP [32] suggests that the CP may be involved in CSF monitoring and antigen presentation [28]. Upon stimulation, immune cells located at the CP can secrete cytokines or infiltrate into the CNS [17]. Accordingly, there is increasing evidence for the involvement of the CP immune component in MS. In the MS mouse model experimental autoimmune encephalomyelitis (EAE), the CP is an important early entry point for immune cells into the CNS [23]. In MS patients, the CSF contains higher numbers of immune cells relative to the CSF in controls [5, 10], which also suggests an increased traversal of immune cells across the BCSFB. In progressive MS immune activation of the CP in a small cohort of SPMS patients has been reported previously [32]. Together, studies suggest that the CP may act as a hub for the regulation of CNS immune homeostasis in MS pathology. On this basis, we here made a detailed assessment of human CP immune cell subsets and their localization within the CP compartments to better understand their role in MS pathogenesis.

We quantitatively and spatially characterized the CP immune cell distribution in progressive MS patients and non-neuroinflammatory controls. We show that granulocytes and T cells, particularly CD8+ T cells, are more abundant in the CP stroma of progressive MS patients compared to controls, but not in the CP epithelium. Moreover, we demonstrate that MHCII+ myeloid cells densely populate the CP of both progressive MS and control cases. Some of these cells appeared to be in close contact with T lymphocytes in the stroma regardless of the disease status, suggesting that antigen presentation is a constitutive process of the CP. Remarkably, B cells and plasma cells were virtually absent in the CP of both progressive MS and controls. Together, this paper highlights the importance of the CP in CNS immune homeostasis, and provides evidence for the involvement of T cells and granulocytes in the CP in the chronic progressive phases of MS.

## Materials and Methods

### Human choroid plexus tissue

Formalin fixed, paraffin embedded CP tissue from the lateral ventricles was obtained from patients with clinically diagnosed, neuro-pathologically confirmed progressive MS (*n* = 16) and non-neuroinflammatory control cases (*n* = 7) by rapid autopsy (Netherlands Brain Bank and Multiple Sclerosis Society Tissue Bank, funded by the Multiple Sclerosis Society of Great Britain and Northern Ireland, registered charity 207,495). All patients and controls, or their next of kin, had given informed consent for autopsy and use of their brain tissue for research purposes. Relevant clinical information was retrieved from the medical records and is summarized in Table 1.

**Table 1:** Clinical data of MS patients and non-neuroinflammatory controls

	Sample	Gender	Age at death	PMD (h)	pH CSF	Brain weight (g)	Diagnosis	Cause of death	Disease duration (y)	Brain Bank
Control	1	m	56	14:00	7.03	1323	Non-demented control	Terminal congestive heart failure	na	NBB
	2	f	78	7:10	6.32	1120	Non-demented control	Legal euthanasia	na	NBB
	3	f	60	8:10	6.58	1310	Non-demented control	Metastasized mammacarcinoma	na	NBB
	4	f	80	7:04	6.2	1450	Non-demented control	Legal euthanasia with metastasis	na	NBB
	5	m	93	7:40	6.2	1155	Dementia with s.i.c.c.	Heart failure	na	NBB
	6	m	73	8:00	5.37	1553	Non-demented control	Invasive fungal infection and bacterial pneumonia	na	NBB
	7	f	95	4:21	6.59	1169	Non-demented control	Ileus, palliative care	na	NBB
Progressive MS	8	m	66	10:55	7.28	na	Multiple sclerosis (PPMS)	Legal euthanasia	25	NBB
	9	m	70	6:55	6.51	1230	Multiple sclerosis (SPMS)	Acute heart failure, *Clostridium difficile* colitis	47	NBB
	10	f	74	7:50	6.4	975	Multiple sclerosis (SPMS)	Legal euthanasia	50	NBB
	11	f	60	9:25	7	1295	Multiple sclerosis (SPMS)	Legal euthanasia with atrial fibrillations and fatigue	22	NBB
	12	m	54	7:55	6.6	1365	Multiple sclerosis (SPMS)	Legal euthanasia	21	NBB
	13	f	57	10:40	6.76	1145	Multiple sclerosis (SPMS)	Legal euthanasia with ataxia	25	NBB
	14	m	82	8:05	6.7	1465	Multiple sclerosis (PPMS)	Pneumonia	44	NBB
	15	m	75	9:10	6.24	1140	Multiple sclerosis (SPMS)	na	na	NBB
	16	f	83	7:40	6.54	1090	Multiple sclerosis (PPMS)	Ovarium carcinoma	34	NBB
	17	f	66	9:30	6.7	1243	Multiple sclerosis (SPMS)	Legal euthanasia	25	NBB
	18	f	49	24:00	6.8	1006	Multiple sclerosis (PPMS)	Multiple sclerosis	na	UK
	19	f	39	15:00	na	998	Multiple sclerosis (SPMS)	Pulmonary embolism, pneumonia	9	UK
	20	m	57	21:00	na	1280	Multiple sclerosis (PPMS)	Multiple sclerosis	na	UK
	21	m	63	10:00	6.52	1614	Multiple sclerosis (PMS, likely PPMS)	Aspiration pneumonia and sepsis; advanced MS	30	NBB
	22	f	61	08:04	6.41	1155	Multiple sclerosis (SPMS)	Urosepsis and hydronepfronis	22	NBB
	23	m	70	05:10	6.82	1181	Multiple sclerosis (SPMS)	Dehydration, decompensation cordis, MS; palliative sedation	21	NBB

*PMD* Post-mortem delay, *f* Female, *m* Male, *na* Not available, *s.i.c.c* Senile involutive cortical changes, *NBB* Netherlands Brain Bank, *UK* Multiple Sclerosis Society Tissue Bank

### Immunohistochemistry

CP tissue was sliced in 5 μm sections, deparaffinized and washed with MilliQ (Millipore). Heat-mediated antigen retrieval was performed in the corresponding buffer (Table 2). Sections were cooled on ice for 30 min and washed with phosphate buffered saline (PBS). Subsequently, sections were blocked with PBS containing 10% normal serum (from the host of the secondary antibody) or bovine serum albumin (BSA, Fraction V, Roche Diagnostics; when using antibodies from multiple hosts) and 0.05% Tween20 (Sigma-Aldrich) for 20 min. Primary antibodies (Table 2) were diluted in PBS containing 1% normal serum or BSA and 0.05% Tween20, and incubated in the dark overnight at 4 °C or for 1 h at room temperature. Then, sections were washed with PBS. Alexa fluorophore-conjugated secondary antibodies (Thermo Fisher Scientific) were diluted in PBS containing 0.05% Tween20 and incubated for 1h at room temperature in the dark*.* After washing with PBS, sections were incubated with Hoechst (33,258, Thermo Fisher Scientific), for nuclear visualization, diluted in PBS to a final concentration of 10 μg/mL for 1 minute in the dark. Sections were washed with PBS, mounted with Mowiol medium and a coverslip (Menzel-Glaser, thickness #1) and stored in the dark at 4 °C until image acquisition.

**Table 2:** Antibody details

Target	Marker	Host	Clonality (clone)	End concentration	Company (catalog number)	Antigen retrieval
APCs	MHCII	Mouse	Monoclonal (LN3)	6.8 μg/mL	Hybridoma	Citrate
B cells	CD19	Rat	Monoclonal (6OMP31)	0.5 μg/mL	Thermo Fisher Scientific (14–0194-82)	Citrate
Basement membrane	Collagen IV	Rabbit	Polyclonal	3.3 μg/mL	Abcam (ab6586)	Tris or citrate
CD4+ T cells	CD4	Rabbit	Monoclonal (EPR6855)	1.1 μg/mL	Abcam (ab133616)	Tris
CD8+ T cells	CD8	Mouse	Monoclonal (C8/144B)	0.157 μg/mL	Dako (M7103)	Tris
Endothelial cells	Biotinylated UEA I Europaeus Agglutinin I (UEA I)			2.0 μg/mL	Vector laboratories (B-1065)	Tris
Granulocytes	CD66b (A647 label)	Mouse	Monoclonal (G10F5)	4.5 μg/mL	Novus Biologicals (NB100-77808AF647)	Citrate
Myeloid cells	Iba1	Goat	Polyclonal	1.0 μg/mL	Abcam (ab5076)	Citrate
Plasma cells	CD138	Mouse	Monoclonal (MI15)	Not available (1:50)	Thermo Fisher Scientific (MA5–12400)	Citrate
T cells	CD3	Mouse	Monoclonal (F7.2.38)	2.8 μg/mL	Dako (M7254)	Tris
T cells	CD3	Rabbit	Polyclonal	3.0 μg/mL	Dako (A0452)	Tris

*APCs* Antigen-presenting cells, Tris: 10 mM Tris / 1 mM EDTA, pH 9; Citrate: 10 mM sodium citrate buffer, pH 6

### Image acquisition and immunostaining scoring

After immunohistochemistry, sections were visualized using a Nikon A1R+ HD confocal galvano scanning laser microscope with 20x magnification and the NIS-Elements software (Nikon). Three image fields per sample displaying characteristic CP morphology were picked based on the basement membrane (collagen IV), while blinded to the other fluorophore channels before final imaging. Because of the abundance of cells in the Iba1/MHCII panel, only one image field was captured. To improve quantification of the Iba1/MHCII and CD66b stainings, a z-stack was created consisting of nine one-micrometre steps. ImageJ was used to process the images, outline the different CP compartments, manually count the cells and quantify the tissue area [1]. This method allows for a quantitative scoring of the immune cells in each of the CP compartments. Imaging and scoring were performed blinded.

For visualization of the interaction between MHCII+ cells with T cells we used a Leica TCS SP8 microscope (Leica Microsystems) and the Leica Application Suite Advanced Fluorescence software (Leica Microsystems). Fifteen non-circulating CD3+ T cells were selected per sample and assessed for interaction with MHCII+ cells.

### Data analysis

Data were analyzed using R version 3.4.2 [29, 30]. For the immune cell quantification, the number of identified immune cells was corrected for analyzed tissue area to calculate cell density. The obtained data were assessed for normality using a Shapiro-Wilk test. In the case of normality, differences between groups were evaluated with a Welch Two Sample t-test. Alternatively, a Wilcoxon rank sum test was applied. Data are reported as the median. Correlations were calculated using the Pearson correlation coefficient. PCA was performed in R using the density of CP (stromal and epithelium-associated) MHCII+ macrophages, MHCII- macrophages, dendritic cells (DCs), total T cells, CD4+ T cells, CD8+ T cells, B cells or plasma cells, granulocytes and the percentage of T cells interacting with MHCII+ cells.

## Results

### Macrophages and dendritic cells densely populate the CP of both progressive MS patients and controls

**Fig. 1:**
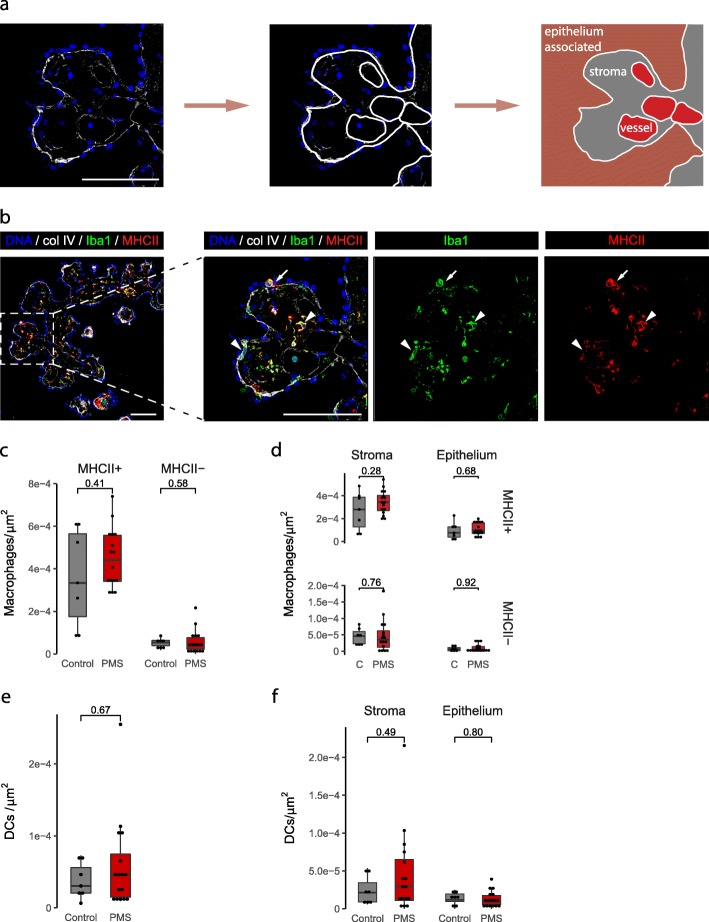
Density of macrophages and DCs is similar in the CP of progressive MS patients and controls**. a** Example of the compartmentalization of the CP tissue. Using the basement membrane as guidance (collagen IV, in white), we discern the stromal and epithelium-associated cells (which together are the CP cells), and exclude the circulating cells located in the vessels. **b** Maximum projection of an image of a CP immunolabeled with Iba1, MHCII and collagen IV. Right panel shows a higher magnification of the image. White arrowheads point to one MHCII+ and one MHCII- macrophage (Iba1+) in the CP stroma; white arrow points to a dendritic cell (DC; Iba1- MHCII+) associated to the epithelium. **c** Density of CP macrophages (MHCII+ and MHCII-) in control and progressive MS (Wilcoxon rank sum test with continuity correction). **d** Density of CP macrophages in the different CP compartments (Welch Two Sample t-test and Wilcoxon rank sum test with continuity correction). **e** Density of CP DCs in control and progressive MS (Wilcoxon rank sum test). **f** Density of CP DCs in the different CP compartments (Wilcoxon rank sum test and Welch Two Sample t-test). Col IV: collagen IV; PMS: progressive MS. Scale bars are 100 μm

In order to characterize the density and location of the immune cell populations within the CP, we performed immunohistochemical analysis of well characterized post-mortem CP of progressive MS cases and non-neuroinflammatory control cases (patient details in Table 1). The visualized CP area was divided into three compartments using the epithelial and endothelial basement membranes (stained by collagen IV) to guide this division. The cellular location was defined as either in the circulation (“vessel”, excluded from the analyses; Additional file 2: Figure S1), in the stromal compartment (“stroma”) or associated to the epithelium (“epithelium”) (Fig. 1a).

To assess the presence of macrophages and dendritic cells (DCs), we immunolabelled CP tissue with Iba1 and MHCII (HLA-DR). The CP of both control and progressive MS cases was densely populated by macrophages (Iba1+ cells) and DCs (defined as Iba1- MHCII+ cells) (Fig. 1b-f). Most of the macrophages were positive for MHCII (Fig. 1c), suggesting their involvement in local antigen presentation; however, a small subset of stromal macrophages was negative for MHCII (Fig. 1b-d). Both macrophages and DCs were mainly located in the stromal compartment (Fig. 1d and f). No differences in macrophage or DC densities were observed between progressive MS and control cases in any of the CP compartments (Fig. 1c-f). In summary, macrophages and DCs densely populate the CP of both controls and progressive MS patients.

### CD8+ T cells are more abundant in progressive MS CP compared to control CP

**Fig. 2:**
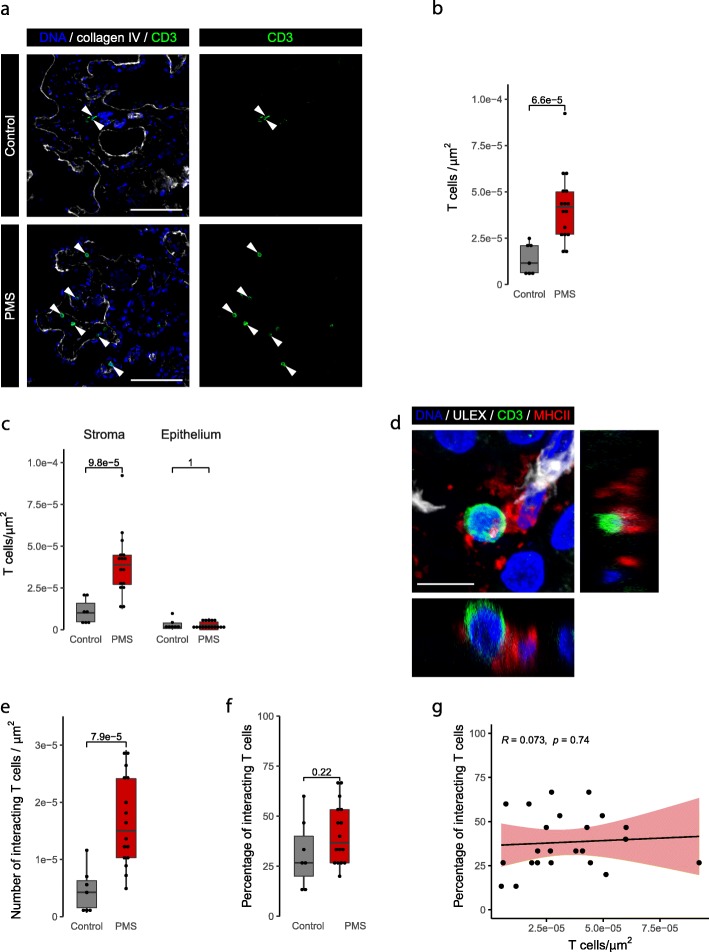
T cell density is higher in the CP stroma of progressive MS patients than in the control CP, and they interact with APCs. **a** Representative images of the control and progressive MS CP immunolabeled with CD3 and collagen IV. White arrowheads point to stromal CD3+ T cells. Scale bars are 100 μm. **b** Density of CP T cells in the CP of control and progressive MS cases (Welch Two Sample t-test). **c** Density of CP T cells in the different CP compartments (Wilcoxon rank sum test). **d** Representative image of a T cell (CD3+, green) in close contact with an APC (MHCII+, red); vessels are visualized with UEA I (white). Maximum projection is accompanied by the orthogonal views. Scale bar is 10 μm. **e** Absolute density of T cells in close contact with MHCII+ APCs in the CP of control and progressive MS patients, calculated by applying the percentage of T cells that were interacting with APCs to the total density of T cells in their respective samples (Welch Two Sample t-test). **f** Percentage of T cells interacting with APCs in the CP of control and progressive MS patients, defined as the CP T cells located directly adjacent to MHCII+ cells (Welch Two Sample t-test). **g** Lack of correlation between interacting T cells and the total CP T cells in each sample (Pearson’s correlation). PMS: progressive MS

As T cells have previously been shown to enter the CNS through the CP in EAE [23], we next assessed the density and distribution of T lymphocytes in the CP of control and progressive MS patients. CD3+ T cells were present in the CP of both control and progressive MS patients (Fig. 2). Importantly, the density of CD3+ T cells was significantly higher in the CP of progressive MS (4.19e-5 cells/μm^2^) compared to control CP (1.15e-5 cells/μm^2^; Fig. 2b). This difference was mainly due to a higher T cell density in the stromal compartment, where the vast majority of T cells were located (Fig. 2c).

Previous research showed that T lymphocytes can infiltrate the mouse CP for re-activation and proliferation [28]. To address this phenomenon in the human CP, we studied the spatial association between CD3+ T cells and MHCII+ APCs in the CP stroma. The presence of T cells adjacent to APCs was commonly observed in both control and progressive MS (Fig. 2d, Additional file 2: Figure S2a-b and Additional file 3: Movie 1). While the calculated absolute number of interacting T cells was higher in progressive MS than in control (Fig. 2e), this was due to the higher density of T cells and there were no differences in the percentage of interacting T cells between the groups (Fig. 2f). Indeed, there was no correlation between the percentage of interacting T cells and the corresponding density of total CP T cells in the MS group (Fig. 2g).

**Fig. 3:**
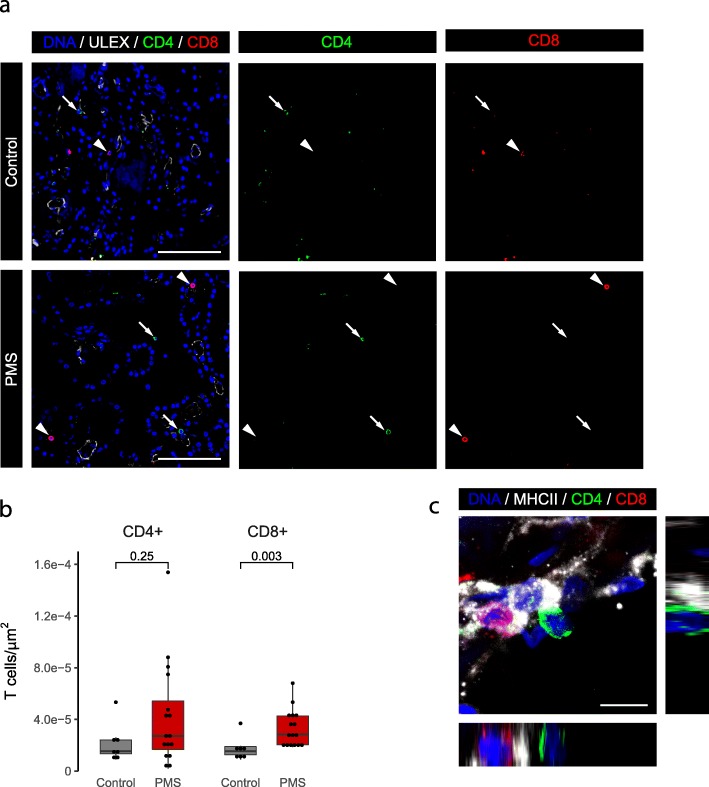
CD8+ T cell density is significantly higher in the CP stroma of progressive MS patients relative to that of controls. **a** Representative images of the progressive MS and control CP immunolabeled with CD4 (green), CD8 (red) and UEA I (white). White arrows point to CD4+ T cells, while white arrowheads point to CD8+ T cells. **b** Density of CP CD4+ and CD8+ T cells in the CP of control and progressive MS patients (Wilcoxon rank sum test). Scale bars are 100 μm. **c** Representative image of a CD4+ T cell (green) and a CD8+ T cell (red) in close contact with an APC (MHCII+, white). Maximum projection is accompanied by the orthogonal views. Scale bar is 10 μm. PMS: progressive MS

To further define the phenotype of these T cells, we analyzed CD4+ helper and CD8+ cytotoxic T cell subsets, with the aid of the endothelial marker UEA I to exclude circulating cells located in the vessels. Both CD4+ and CD8+ T cells were present in the CP of all cases, but only CD8+ T cell density was significantly higher in progressive MS patients 2.83e-5 cells/μm^2^) relative to controls (1.53e-5 cells/μm^2^; Fig. 3a and b). Both CD4+ and CD8+ T cells were found in close contact with APCs (Fig. 3c, Additional file 2: Figure S2c). Together, our data indicate that T cells are present in the CP, where they interact with APCs in both controls and progressive MS patients, and that there is a higher density of CD8+ T cells in progressive MS patients relative to controls.

### B cells and plasma cells are virtually absent from the CP

**Fig. 4:**
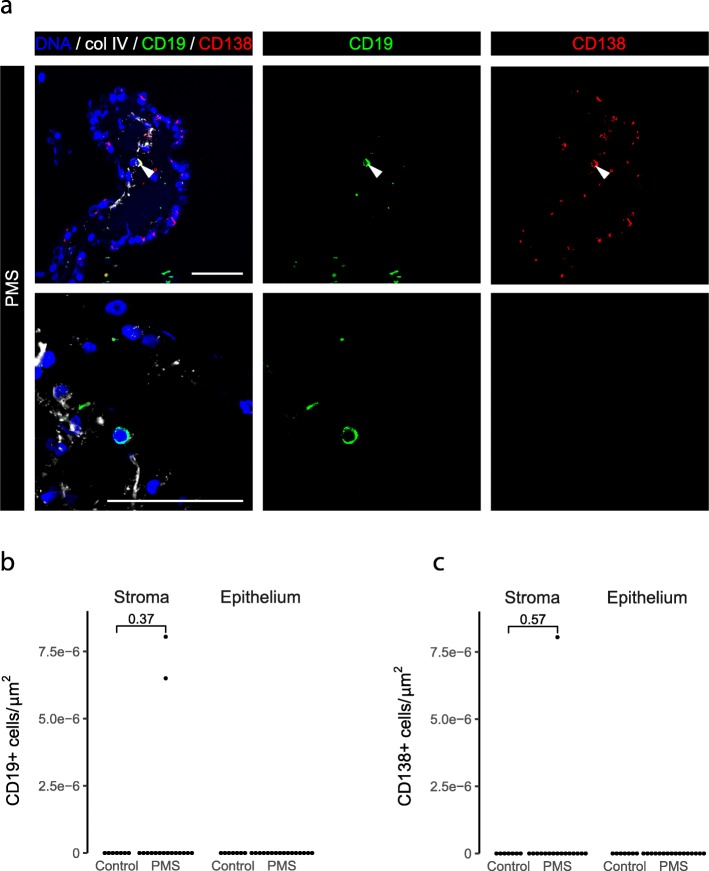
B and plasma cells are virtually absent from the CP. **a** Examples of CD19+ and CD138+ cells in the CP of progressive MS patients; basement membrane was immunolabeled with collagen IV. White arrowhead in the top panel points to a CD19+ CD138+ cell; in the lower panel, a CD19+ B cell is seen. **b** Density of CP CD19+ cells in the different CP compartments (Wilcoxon rank sum test with continuity correction). **c** Density of CP CD138+ cells in the different CP compartments (Wilcoxon rank sum test with continuity correction). PMS: progressive MS. Scale bars are 50 μm

B cells are implicated in the pathogenesis of progressive MS, as demonstrated by the efficacy of CD20-targeted therapies [7, 8, 13, 25]. In the meninges, B cells and plasma cells are present in the follicle-like structures found in some progressive MS patients [22, 26], and there are more B cells in the CSF of progressive MS patients relative to controls [10]. Thus, we set out to investigate whether B cells and plasma cells are present in the CP of progressive MS patients and controls. In most MS patients and all controls, we did not observe any B cells (marked by CD19) and/or plasma cells (marked by CD138). In one progressive MS patient, a double positive cell for CD19 and CD138 was identified in the stroma (Fig. 4). Only one CD19+ CD138- B cell was detected in our patient cohort (Fig. 4a-b). In sum, B cells and plasma cells are virtually absent from the CP of both progressive MS patients and controls.

### Granulocytes are more abundant in the CP of progressive MS patients compared to control CP

**Fig. 5:**
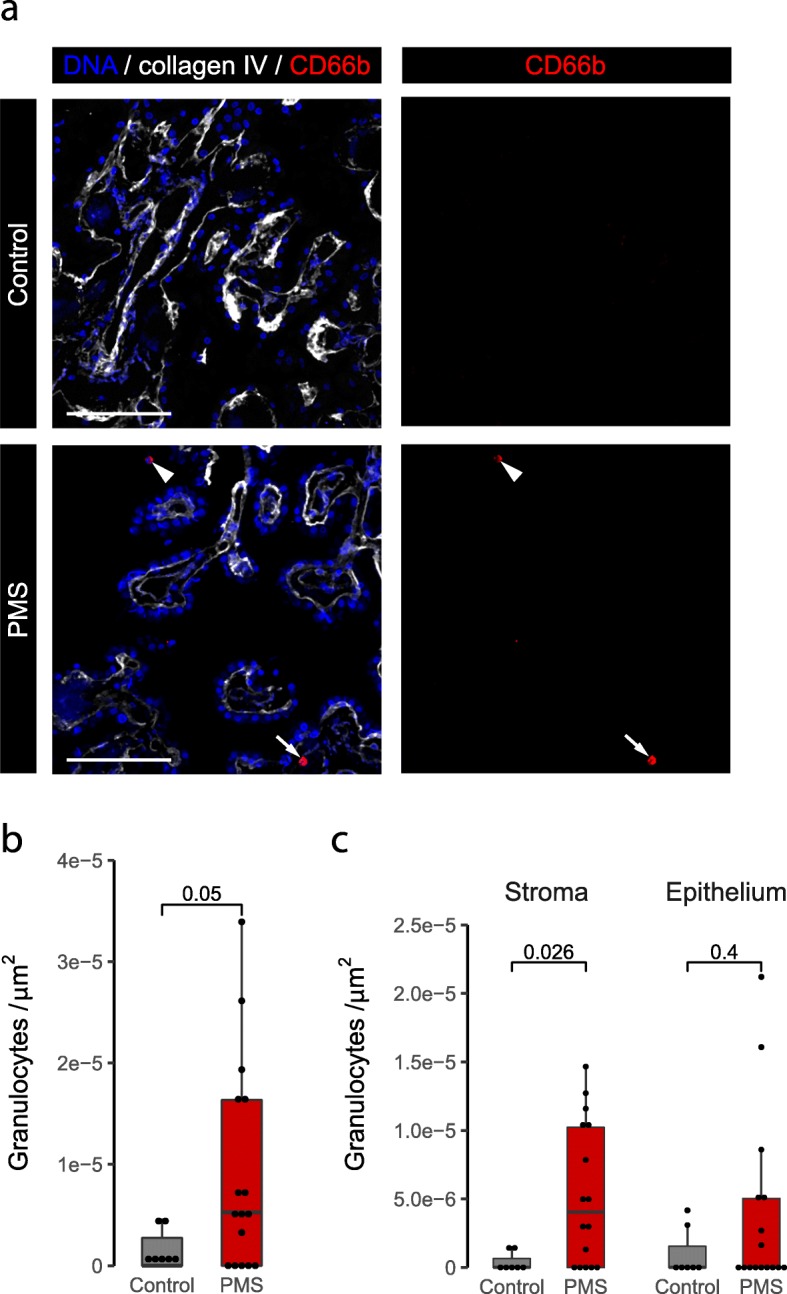
Granulocyte density is higher in the CP of progressive MS patients relative to the control CP. **a** Maximum projection image of a progressive MS and a control CP immunolabeled with CD66b (red) and collagen IV (white). White arrowhead points to a granulocyte associated with the epithelium and white arrow points to a stromal granulocyte in the progressive MS case. **b** Density of CP granulocytes in the CP of control and progressive MS cases (Wilcoxon rank sum test with continuity correction). **c** Density of CP granulocytes in the different CP compartments (Wilcoxon rank sum test with continuity correction). PMS: progressive MS. Scale bars are 100 μm

The role of granulocytes has been underappreciated in MS pathology [33]. We studied the presence of granulocytes in the CP using the marker CD66b. As expected due to their abundance in blood, most of the granulocytes were detected in the vessels (Additional file 2: Figure S1f). In progressive MS patients, the density of non-circulating granulocytes was significantly higher than in controls (5.26e-6 cells/μm^2^ vs 0 cells/μm^2^; Fig. 5b). This difference was mainly due to the stromal compartment (Fig. 5c), although in the progressive MS CP epithelium there was also a trend to a higher density of granulocytes compared to the control. Preliminary stainings show that most of the granulocytes were neutrophils, as shown by their expression of elastase (Additional file 2: Figure S3). These findings indicate that granulocyte infiltration is apparent in the CP of progressive MS patients.

## Discussion

**Fig. 6:**
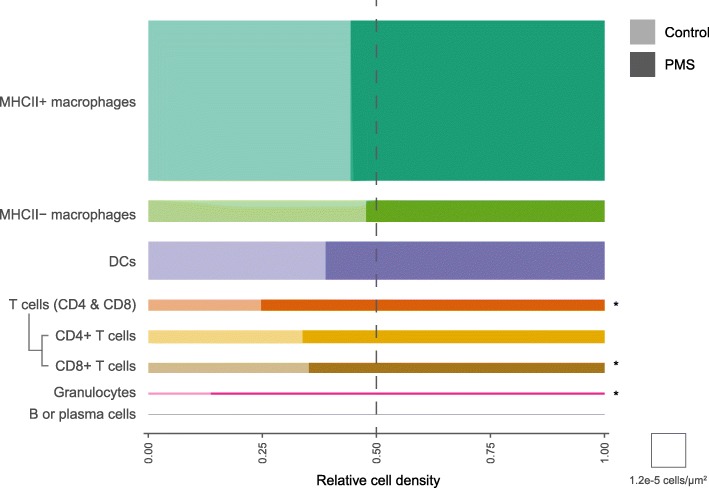
Graphical summary of the immune cell populations in the CP of progressive MS patients and controls. X axis shows the density of each immune cell population in control and progressive MS CP. The area of each bar represents the mean density of the corresponding cell type and disease status. The bar length represents the relative contribution of each disease status to the total cells of a particular subset. Significant alterations in the density of cell populations between progressive MS and control cases are represented with an asterisk. Fold change could not be calculated for B or plasma cells due to the 0 values in the control cases. PMS: progressive MS

By using human post-mortem CP tissue in combination with immunohistochemistry and confocal microscopy, we here provide a comprehensive overview of immune cell populations present in the CP of non-neuroinflammatory controls and progressive MS cases, both in terms of abundance and of their location within the different CP compartments (summarized in Fig. 6 and Additional file 1: Table S1). For the first time, we show that both CD8+ T cells and granulocytes are more abundant in the CP stroma of progressive MS cases compared to controls. Around one third of the T cells appeared to be in close contact with APCs in the CP regardless of disease status, suggesting that local antigen presentation is a constitutive event in the CP. Furthermore, we show that macrophages, particularly those expressing MHCII, and DCs are abundant in CP of both MS patients and controls, with the majority located in the stroma but also associated with the epithelium. In contrast, B and plasma cells were rarely observed in both the MS and control CP.

The CP is a strategically located, highly vascularized structure within the brain ventricles with CNS homeostatic properties. The CP acts as a bidirectional immunosurveillance system by means of the BCSFB [28]. To date, relatively little is known about the immune cell populations in the human CP under normal and inflammatory conditions. We here provide a detailed characterization of the main immune cell populations in the CP of controls and progressive MS patients, which can be a resource for researchers in the field of brain barriers and MS, while helping to better understand the inflammatory processes in the progressive phases of MS.

T cells play a central role in the pathogenesis of MS [15, 19]. Here, we observed that the CP presents a higher density of T cells in MS patients relative to controls, in line with a previous study of Vercellino and co-workers [32]. We further demonstrated that T lymphocytes are also present in control individuals, as reported before [16]. In contrast, another study found no T cells in control CP [32], which may be explained by a small number of control cases used in that study. T cells are predominantly localized in the stromal compartment of the CP. Stromal T lymphocytes may be infiltrated from the periphery into the CNS [23], migrated from the CSF into the CP for re-activation [28] or remain in the CP as tissue resident T cells. The relative scarcity of epithelium associated T cells, together with the lack of changes in T cell numbers in the CSF of progressive MS patients [10], supports the view that T cells in the CP of progressive MS are restricted to the stromal compartment and do not cross the BCSFB at the CP. However, we cannot exclude the possibility that subtle differences in epithelial T cell density are missed due to the brevity of the migration process and the relative small size of the epithelial compartment, which result in a low chance of detecting them. CD8+ T cells are the predominant T cell subtype in MS lesions [3] and we observed accumulation of CD8+ T cells in the progressive MS CP. While CD8+ T cells within the brain have detrimental effects on CNS cells, those restricted to the CNS borders may exert immunosuppressive effects [14, 15, 31], or simply reside as tissue memory T cells as a result of previous inflammatory processes.

The role of granulocytes in MS pathology has been underappreciated as they are rarely reported in MS lesions [2, 33]. In RRMS, the number of neutrophils in the CSF decreases with disease duration [18]; in contrast, PPMS subjects have more granulocytes than RRMS patients in the CSF [10]. Interestingly, our unpublished findings showed that the neutrophil chemoattractant gene *CXCL2* is upregulated in the CP of progressive MS patients. Here, we observed more granulocytes in the CP of progressive MS patients compared to the CP from controls. Because granulocytes have a particularly short lifespan, our results point to a continuous infiltration from the peripheral circulation at this chronic stage of the disease. Although granulocytes are typically regarded as innate immune cells detrimental for tissue repair, the release of reactive oxygen species and degranulation can also have a regulatory effect in the adaptive immune system. As such, neutrophils exert an immunosuppressive effect on T cell responses to myelin [34]. Whether they play a similar role in the progressive MS CP and thereby restrict T cell infiltration at the CP needs to be further elucidated.

Macrophages and DCs are the predominant immune cell type in the CP stroma. Their antigen-presenting capacity is illustrated by the expression of MHCII on their surface. In addition to their stromal location, we observed that macrophages and DCs were associated with the CP epithelium, either protruding among epithelial cells or associated with the apical surface of the epithelium (known as Kolmer cells or epiplexus macrophages), which confirms and extends earlier observations [11, 27, 32]. These epithelium-associated immune cells at the BCSFB are likely a bridge between the peripheral and CNS immune systems [11, 12]. Thus, the CP may have constitutive functions in CNS homeostasis through this niche of macrophages and DCs, such as local presentation of CNS antigens.

Our study revealed the virtual absence of B cells and plasma cells in the CP. Importantly, our results are in line with previous research, which described very rare CD138+ plasma cells and an absence of CD20+ B cells [32]. Thus, the CP does not appear to be an initial CNS-entry site for B cells during the progressive phase of MS. Instead, B cells present in the meninges of progressive MS patients [22, 26] may represent the primary source of infiltrating B cells into the CSF.

This study is constrained by the availability of human CP tissue. Thus, the variability in the immune populations between donors (Additional file 2: Figure S4) may have hindered the detection of subtle differences. It would be interesting to explore the CP immune cell composition of RRMS patients, however we could not obtain high quality CP samples from RRMS patients. Despite the heterogeneity in immune cell densities among the patients, we did not find differences between PPMS and SPMS patients (Additional file 2: Figure S5). Although PPMS and SPMS present different disease courses, in both progressive forms of MS neurodegeneration predominates over acute inflammation. Accordingly, a moderately inflamed status is seen in the CP of both PPMS and SPMS patients. Although we tried to use consensus cellular markers, no single marker can identify human DCs. By defining DCs as MHCII+ Iba1-, we could not exclude other APCs, such as B cells or activated T cells. However, considering the scarcity of B cells and the lack or subtle expression of MHCII by T cells in the CP, we expect this error to be negligible. Lastly, the use of fixed post-mortem material provides only a snapshot of a dynamic process that cannot fully reflect the progression of the disease.

In summary, this study provides a detailed characterization of the density and location of immune cell populations present in the human CP, as well as alterations thereof in progressive MS. The presence of immune cells in non-neuroinflammatory controls highlights the role of the CP in immune surveillance and homeostasis. Further, we provide insight into the involvement of the inflammatory component of the CP in progressive MS. Particularly, the abundance of T cells and granulocytes at the CP in progressive MS patients implicate both the adaptive and innate immune systems in the chronic progressive phases of MS. However, their restriction to the stromal compartment argues against the CP as a major entry route of immune cells into the CNS during progressive MS. Future research is warranted to unravel the functional consequences of the immune changes in the CP, and how these may in turn affect the CNS of MS patients.

## Supplementary information

**Additional file 1: Table S1**. Density of CP (stromal and epithelium-associated) immune cell populations in control and progressive MS patients.

**Additional file 2: Figure S1.** Immune cells located in the vessel compartment of the CP of progressive MS patients and controls. **a)** Iba1+ cells and their MHCII expression. **b)** Iba1- MHCII+ cells. **c)** CD3+ T cells. **d)** CD19+ B cells. **e)** CD138+ plasma cells. **f)** CD66b + granulocytes (Wilcoxon rank sum test with continuity correction). C: control; PMS: progressive MS. **Figure S2.** T cells in close contact with APCs in the CP stroma. **a)** Representative images of the CP immunolabeled with CD3 (green) and MHCII (red); vessels are visualized with UEA I (white). White arrowheads point to a CD3+ T cell in close contact with an MHCII+ APC (left panel), and to a non-interacting T cell (right panel). **b)** On the top panel, three T cells can be seen: one is not interacting with any MHCII+ cell (white arrow), while the other two are in close contact with MHCII+ cells (white arrowheads). The middle and lower panels show higher magnification of T lymphocytes interacting with APCs. **c)** Percentage of CD4+ T cells and CD8+ T cells interacting with APCs in the CP of control and progressive MS patients, defined as the T cells located directly adjacent to MHCII+ cells (Wilcoxon rank sum test with continuity correction). Scale bar is 10 μm. **Figure S3.** Most granulocytes in the CP are neutrophils. Representative images of one CP section immunolabeled with CD66b (red) and elastase (green). Maximum projection image. White arrowheads point to CD66b + elastase+ neutrophils. Scale bars are 50 μm. **Figure S4.** PCA plot of the samples used in this study, showing standardized principal components 1 and 2. Axes show the percentage of variance explained by each principal component. Variables included in the analysis: density of CP MHCII+ macrophages, MHCII- macrophages, DCs, total T cells, CD4+ and CD8+ T cells, percentage of T cells interacting with MHCII+ cells, B or plasma cells and granulocytes. PC: principal component; PMS: progressive MS. **Figure S5.** PPMS and SPMS patients present similar non-circulating (stromal and epithelium-associated) immune cell subsets in the CP. **a)** Density of non-circulating CD3+ T cells in PPMS and SPMS patients (Welch Two Sample t-test). **b)** Ratio of non-circulating CD4+ vs CD8+ T cells in PPMS and SPMS patients (Welch Two Sample t-test). **c)** Density of non-circulating MHCII+ macrophages in PPMS and SPMS patients (Welch Two Sample t-test). **d)** Density of non-circulating MHCII- macrophages in PPMS and SPMS patients (Welch Two Sample t-test). **d)** Density of non-circulating Iba1-MHCII+ DCs in PPMS and SPMS patients (Wilcoxon rank sum test). **e)** Density of non-circulating granulocytes in PPMS and SPMS patients (Wilcoxon rank sum test). PPMS: Primary Progressive MS; SPMS: Secondary Progressive MS

**Additional file 3: Movie 1.** Example of a T cell (CD3+, green) adjacent to an APC (MHCII+, red) in the CP. Nuclei are in blue and vessels are marked with UEA I in white.

## Data Availability

The datasets used and/or analyzed during the current study are available from the corresponding author on reasonable request.

## Abbreviations

BBB: Blood brain barrier
BCSFB: Blood-cerebrospinal fluid barrier
BSA: Bovine serum albumin
CNS: Central nervous system
CP: Choroid plexus
CSF: Cerebrospinal fluid
DC: Dendritic cell
EAE: Experimental Autoimmune Encephalomyelitis
MS: Multiple sclerosis
PBS: Phosphate buffered saline
PCA: Principal component analysis
PPMS: Primary progressive multiple sclerosis
RRMS: Relapsing-remitting multiple sclerosis
SPMS: Secondary progressive multiple sclerosis

## References

[1] Abràmoff MD, Magalhães PJ, Ram SJ (2004). Image processing with ImageJ. Biophotonics Int.

[2] Aube B, Levesque SA, Pare A, Chamma E, Kebir H, Gorina R, Lecuyer MA, Alvarez JI, De Koninck Y, Engelhardt B, Prat A, Cote D, Lacroix S (2014). Neutrophils mediate blood-spinal cord barrier disruption in demyelinating neuroinflammatory diseases. J Immunol.

[3] Booss J, Esiri MM, Tourtellotte WW, Mason DY (1983). Immunohistological analysis of T lymphocyte subsets in the central nervous system in chronic progressive multiple sclerosis. J Neurol Sci.

[4] Carrithers MD, Visintin I, Viret C, Janeway CS (2002). Role of genetic background in P selectin-dependent immune surveillance of the central nervous system. J Neuroimmunol.

[5] Cepok S, Jacobsen M, Schock S, Omer B, Jaekel S, Boddeker I, Oertel WH, Sommer N, Hemmer B (2001). Patterns of cerebrospinal fluid pathology correlate with disease progression in multiple sclerosis. Brain.

[6] Choi SR, Howell OW, Carassiti D, Magliozzi R, Gveric D, Muraro PA, Nicholas R, Roncaroli F, Reynolds R (2012). Meningeal inflammation plays a role in the pathology of primary progressive multiple sclerosis. Brain.

[7] Cross AH, Stark JL, Lauber J, Ramsbottom MJ, Lyons JA (2006). Rituximab reduces B cells and T cells in cerebrospinal fluid of multiple sclerosis patients. J Neuroimmunol.

[8] del Pilar MM, Cravens PD, Winger R, Kieseier BC, Cepok S, Eagar TN, Zamvil SS, Weber MS, Frohman EM, Kleinschmidt-DeMasters BK (2009). Depletion of B lymphocytes from cerebral perivascular spaces by rituximab. Arch Neurol.

[9] Dutta R, Trapp BD (2014). Relapsing and progressive forms of multiple sclerosis: insights from pathology. Curr Opin Neurol.

[10] Han S, Lin YC, Wu T, Salgado AD, Mexhitaj I, Wuest SC, Romm E, Ohayon J, Goldbach-Mansky R, Vanderver A, Marques A, Toro C, Williamson P, Cortese I, Bielekova B (2014). Comprehensive immunophenotyping of cerebrospinal fluid cells in patients with neuroimmunological diseases. J Immunol.

[11] Hanly A, Petito CK (1998). HLA-DR-positive dendritic cells of the normal human choroid plexus: a potential reservoir of HIV in the central nervous system. Hum Pathol.

[12] Hatterer E, Touret M, Belin MF, Honnorat J, Nataf S (2008). Cerebrospinal fluid dendritic cells infiltrate the brain parenchyma and target the cervical lymph nodes under neuroinflammatory conditions. PLoS One.

[13] Hauser SL, Waubant E, Arnold DL, Vollmer T, Antel J, Fox RJ, Bar-Or A, Panzara M, Sarkar N, Agarwal S, Langer-Gould A, Smith CH, Group HT (2008). B-cell depletion with rituximab in relapsing-remitting multiple sclerosis. N Engl J Med.

[14] Jiang H, Zhang SI, Pernis B (1992). Role of CD8+ T cells in murine experimental allergic encephalomyelitis. Science.

[15] Johnson AJ, Suidan GL, McDole J, Pirko I (2007). The CD8 T cell in multiple sclerosis: suppressor cell or mediator of neuropathology?. Int Rev Neurobiol.

[16] Kivisakk P, Mahad DJ, Callahan MK, Trebst C, Tucky B, Wei T, Wu L, Baekkevold ES, Lassmann H, Staugaitis SM, Campbell JJ, Ransohoff RM (2003). Human cerebrospinal fluid central memory CD4+ T cells: evidence for trafficking through choroid plexus and meninges via P-selectin. Proc Natl Acad Sci U S A.

[17] Korin B, Ben-Shaanan TL, Schiller M, Dubovik T, Azulay-Debby H, Boshnak NT, Koren T, Rolls A (2017). High-dimensional, single-cell characterization of the brain's immune compartment. Nat Neurosci.

[18] Kostic M, Dzopalic T, Zivanovic S, Zivkovic N, Cvetanovic A, Stojanovic I, Vojinovic S, Marjanovic G, Savic V, Colic M (2014). IL-17 and glutamate excitotoxicity in the pathogenesis of multiple sclerosis. Scand J Immunol.

[19] Lassmann H, Bruck W, Lucchinetti CF (2007). The immunopathology of multiple sclerosis: an overview. Brain Pathol.

[20] Lassmann H, van Horssen J, Mahad D (2012). Progressive multiple sclerosis: pathology and pathogenesis. Nat Rev Neurol.

[21] Luchetti S, Fransen NL, van Eden CG, Ramaglia V, Mason M, Huitinga I (2018). Progressive multiple sclerosis patients show substantial lesion activity that correlates with clinical disease severity and sex: a retrospective autopsy cohort analysis. Acta Neuropathol.

[22] Magliozzi R, Howell O, Vora A, Serafini B, Nicholas R, Puopolo M, Reynolds R, Aloisi F (2007). Meningeal B-cell follicles in secondary progressive multiple sclerosis associate with early onset of disease and severe cortical pathology. Brain.

[23] Reboldi A, Coisne C, Baumjohann D, Benvenuto F, Bottinelli D, Lira S, Uccelli A, Lanzavecchia A, Engelhardt B, Sallusto F (2009). C-C chemokine receptor 6-regulated entry of TH-17 cells into the CNS through the choroid plexus is required for the initiation of EAE. Nat Immunol.

[24] Reich DS, Lucchinetti CF, Calabresi PA (2018). Multiple Sclerosis. N Engl J Med.

[25] Sabatino JJ, Probstel AK, Zamvil SS (2019). B cells in autoimmune and neurodegenerative central nervous system diseases. Nat Rev Neurosci.

[26] Serafini B, Rosicarelli B, Magliozzi R, Stigliano E, Aloisi F (2004). Detection of ectopic B-cell follicles with germinal centers in the meninges of patients with secondary progressive multiple sclerosis. Brain Pathol.

[27] Serot JM, Foliguet B, Bene MC, Faure GC (1997). Ultrastructural and immunohistological evidence for dendritic-like cells within human choroid plexus epithelium. Neuroreport.

[28] Strominger I, Elyahu Y, Berner O, Reckhow J, Mittal K, Nemirovsky A, Monsonego A (2018). The choroid plexus functions as a niche for T-cell stimulation within the central nervous system. Front Immunol.

[29] Team R (2015). RStudio: integrated development for R.

[30] Team RC (2017). R: a language and environment for statistical com-puting.

[31] Tennakoon DK, Mehta RS, Ortega SB, Bhoj V, Racke MK, Karandikar NJ (2006). Therapeutic induction of regulatory, cytotoxic CD8+ T cells in multiple sclerosis. J Immunol.

[32] Vercellino M, Votta B, Condello C, Piacentino C, Romagnolo A, Merola A, Capello E, Mancardi GL, Mutani R, Giordana MT, Cavalla P (2008). Involvement of the choroid plexus in multiple sclerosis autoimmune inflammation: a neuropathological study. J Neuroimmunol.

[33] Woodberry Tonia, Bouffler Sophie, Wilson Alicia, Buckland Rebecca, Brüstle Anne (2018). The Emerging Role of Neutrophil Granulocytes in Multiple Sclerosis. Journal of Clinical Medicine.

[34] Zehntner SP, Brickman C, Bourbonniere L, Remington L, Caruso M, Owens T (2005). Neutrophils that infiltrate the central nervous system regulate T cell responses. J Immunol.

