# Correction to: Inflammation of the choroid plexus in progressive multiple sclerosis: accumulation of granulocytes and T cells

**DOI:** 10.1186/s40478-020-00899-5

**Published:** 2020-02-26

**Authors:** Sabela Rodríguez-Lorenzo, Julia Konings, Susanne van der Pol, Alwin Kamermans, Sandra Amor, Jack van Horssen, Maarten E. Witte, Gijs Kooij, Helga E. de Vries

**Affiliations:** 1 Department of Molecular Cell Biology and Immunology, MS Center Amsterdam, Amsterdam Neuroscience, AmsterdamUMC, Vrije Universiteit Amsterdam, De Boelelaan, 1117 Amsterdam, The Netherlands; 2 Department of Pathology, MS center Amsterdam, Amsterdam UMC, Vrije Universiteit Amsterdam, Amsterdam, The Netherlands; 3 Centre for Neuroscience and Trauma, Blizard Institute, Barts and the London School of Medicine and Dentistry, Queen Mary University of London, London, UK; 4 Medical Biochemistry, Amsterdam Cardiovascular Sciences, Amsterdam UMC, University of Amsterdam, Meibergdreef 9, Amsterdam, 1105 AZ The Netherlands

**Correction to: Acta Neuropathol Commun**

**https://doi.org/10.1186/s40478-020-0885-1**

The original publication of this article [1] contained an incorrect author name. The correct and incorrect information is shown in this correction article. The original article has been updated.

The incorrect author name was published as:

- Maarten Witte

The correct author is:

- Maarten E Witte
