# Supplementary material for: Inflammation of the choroid plexus in progressive multiple sclerosis: accumulation of granulocytes and T cells
Source: Acta Neuropathol Commun. 2020 Feb 3;8:9. doi: 10.1186/s40478-020-0885-1 (PMC6998074; doi:10.1186/s40478-020-0885-1)
Supplement: Supplementary file 1 — Additional file 1: Table S1. Density of CP (stromal and epithelium-associated) immune cell populations in control and progressive MS patients. [file 40478_2020_885_MOESM1_ESM.docx]

Additional file 1: Table S1. Density of CP (stromal and epithelium-associated) immune cell populations in control and progressive MS patients.

|  | **Control** | | **Progressive MS** | |
| --- | --- | --- | --- | --- |
|  | **Median** | **IQR** | **Median** | **IQR** |
| **MHCII+ macrophages** | 3.34E-04 | 3.89E-04 | 4.40E-04 | 2.16E-04 |
| **MHCII- macrophages** | 5.33E-05 | 2.41E-05 | 3.84E-05 | 5.48E-05 |
| **DCs** | 6.09E-05 | 7.09E-05 | 9.18E-05 | 1.20E-04 |
| **T cells (CD4 & CD8)** | 1.15E-05 | 1.46E-05 | 4.18E-05 | 2.28E-05 |
| **CD4+ T cells** | 1.53E-05 | 1.07E-05 | 2.72E-05 | 3.76E-05 |
| **CD8+ T cells** | 1.53E-05 | 6.29E-06 | 2.83E-05 | 2.24E-05 |
| **Granulocytes** | 0.00E+00 | 2.74E-06 | 5.26E-06 | 1.64E-05 |
| **B or plasma cells** | 0.00E+00 | 0.00E+00 | 0.00E+00 | 0.00E+00 |

IQR: Interquartile range
