# Supplementary figures and images for: Inflammation of the choroid plexus in progressive multiple sclerosis: accumulation of granulocytes and T cells
Source: Acta Neuropathol Commun. 2020 Feb 3;8:9. doi: 10.1186/s40478-020-0885-1 (PMC6998074; doi:10.1186/s40478-020-0885-1)

Figure S1

a

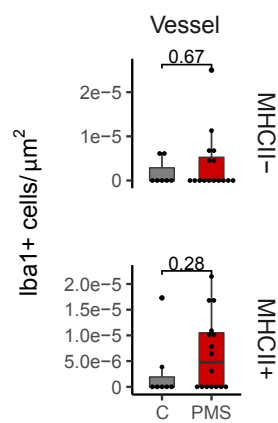

b

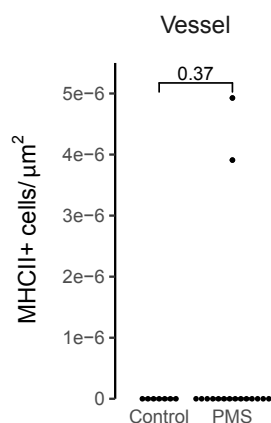

c

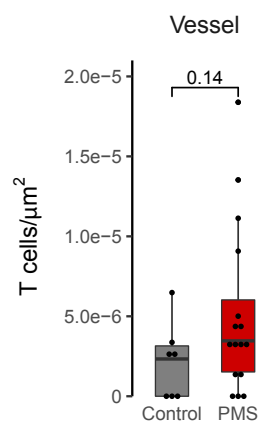

d

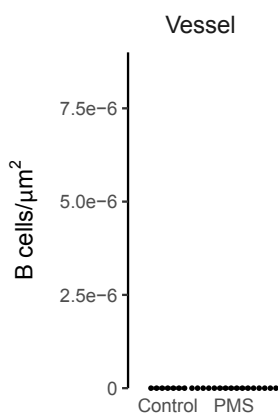

e

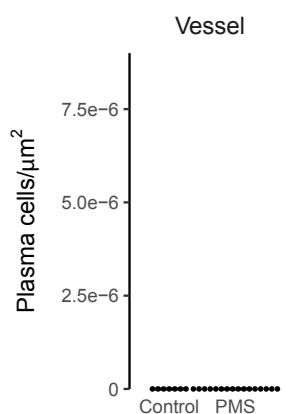

f

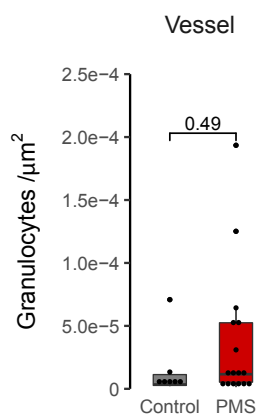

Figure S2

a

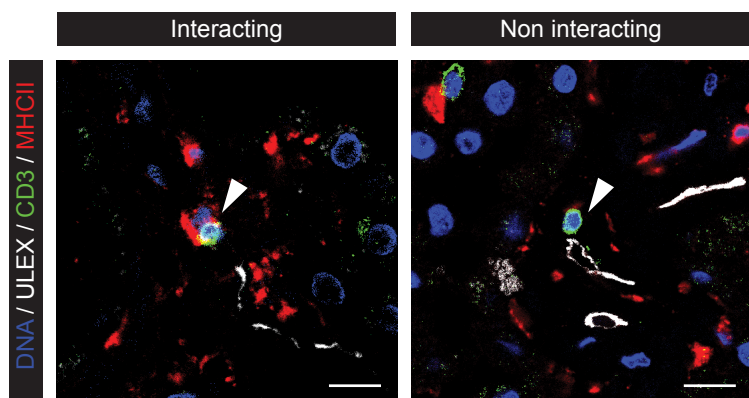

b

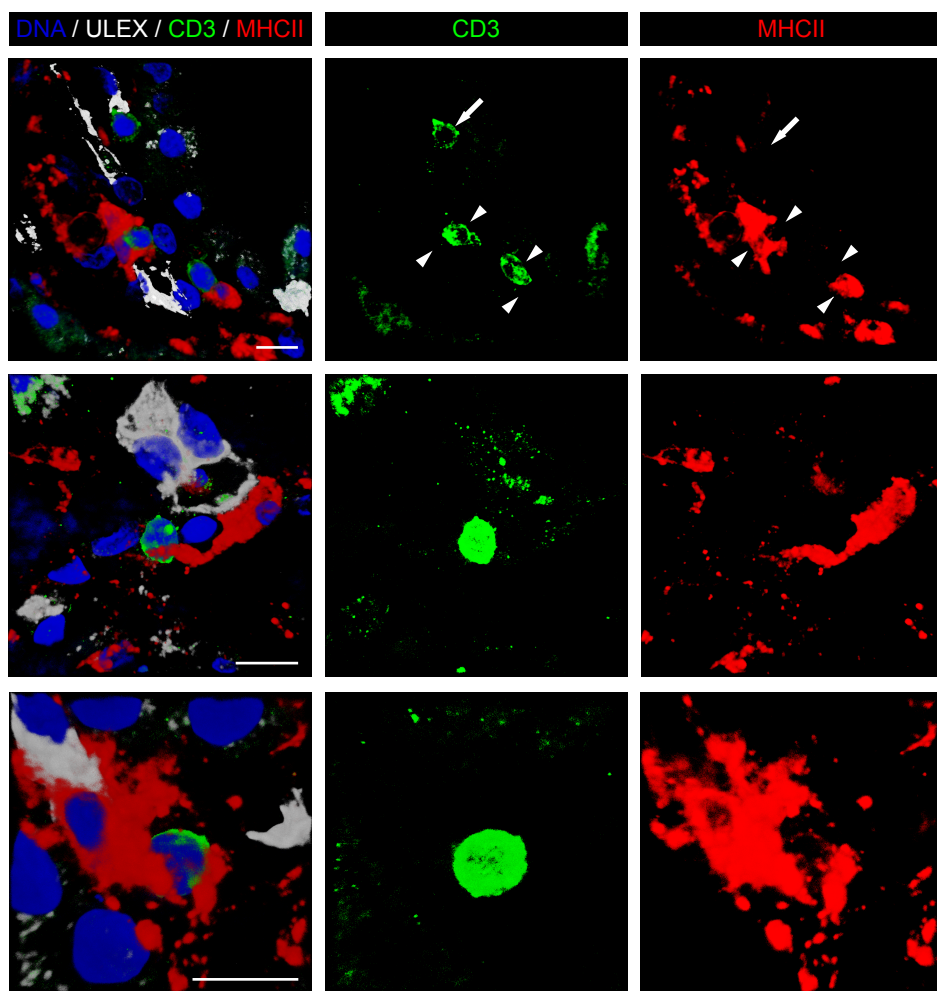

c

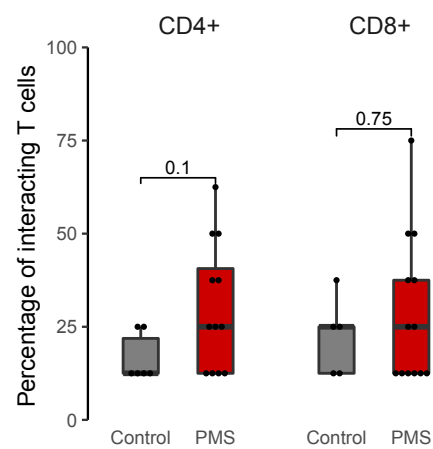

Figure S3

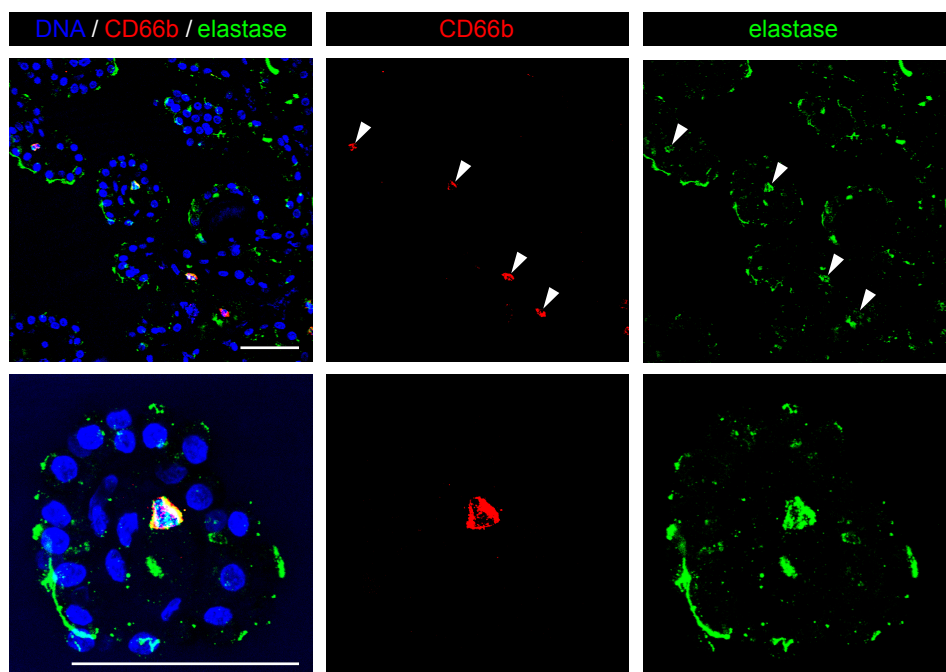

Figure S4

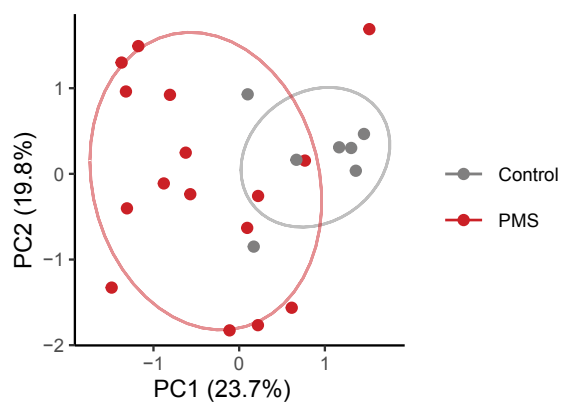

Figure S5

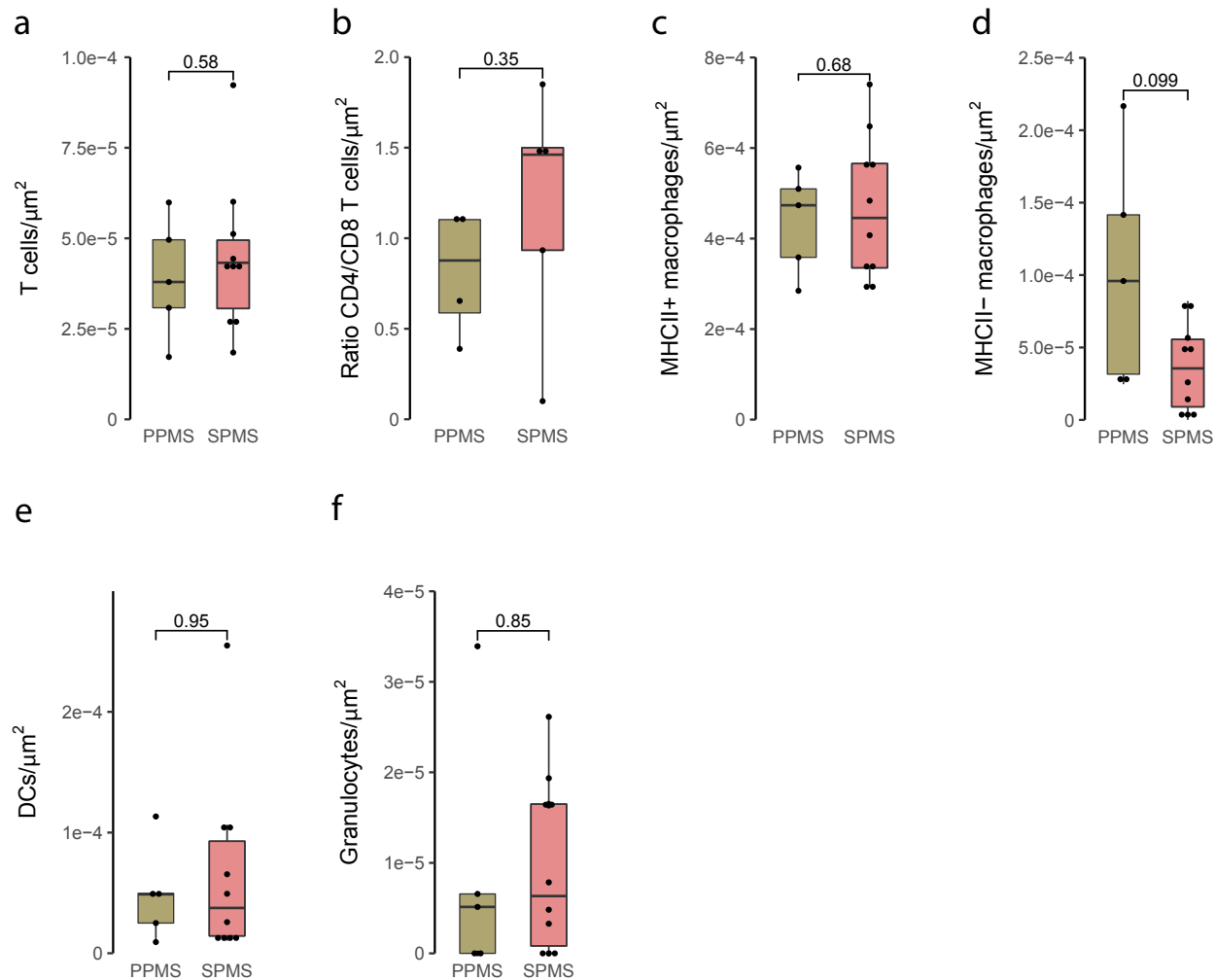

Supplement: Supplementary file 2 — Additional file 2: Figure S1. Immune cells located in the vessel compartment of the CP of progressive MS patients and controls. a) Iba1+ cells and their MHCII expression. b) Iba1- MHCII+ cells. c) CD3+ T cells. d) CD19+ B cells. e) CD138+ plasma cells. f) CD66b + granulocytes (Wilcoxon rank sum test with continuity correction). C: control; PMS: progressive MS. Figure S2. T cells in close contact with APCs in the CP stroma. a) Representative images of the CP immunolabeled with CD3 (green) and MHCII (red); vessels are visualized with UEA I (white). White arrowheads point to a CD3+ T cell in close contact with an MHCII+ APC (left panel), and to a non-interacting T cell (right panel). b) On the top panel, three T cells can be seen: one is not interacting with any MHCII+ cell (white arrow), while the other two are in close contact with MHCII+ cells (white arrowheads). The middle and lower panels show higher magnification of T lymphocytes interacting with APCs. c) Percentage of CD4+ T cells and CD8+ T cells interacting with APCs in the CP of control and progressive MS patients, defined as the T cells located directly adjacent to MHCII+ cells (Wilcoxon rank sum test with continuity correction). Scale bar is 10 μm. Figure S3. Most granulocytes in the CP are neutrophils. Representative images of one CP section immunolabeled with CD66b (red) and elastase (green). Maximum projection image. White arrowheads point to CD66b + elastase+ neutrophils. Scale bars are 50 μm. Figure S4. PCA plot of the samples used in this study, showing standardized principal components 1 and 2. Axes show the percentage of variance explained by each principal component. Variables included in the analysis: density of CP MHCII+ macrophages, MHCII- macrophages, DCs, total T cells, CD4+ and CD8+ T cells, percentage of T cells interacting with MHCII+ cells, B or plasma cells and granulocytes. PC: principal component; PMS: progressive MS. Figure S5. PPMS and SPMS patients present similar non-circulating (st [file 40478_2020_885_MOESM2_ESM.pdf]
